# Bibliometric mapping of intensive care nurses’ wellbeing: development and application of the new iAnalysis model

**DOI:** 10.1186/s12912-019-0343-1

**Published:** 2019-06-03

**Authors:** Rebecca J. Jarden, Ajit Narayanan, Margaret Sandham, Richard J. Siegert, Jane Koziol-McLain

**Affiliations:** 1 Present Address: Department of Nursing, Melbourne School of Health Sciences, 161 Barry St, Carlton, Victoria 3053 Australia; 2 School of Clinical Sciences, Auckland University of Technology (AUT), North Shore Campus, 90 Akoranga Drive, Northcote, Auckland, 0627 New Zealand; 3 School of Engineering, Computing and Mathematical Sciences (D-75), Auckland University of Technology, AUT Tower, 2-14 Wakefield Street, Auckland, 1010 New Zealand; 4 School of Clinical Sciences and School of Public Health and Psychosocial Studies, Auckland University of Technology (AUT), North Shore Campus, 90 Akoranga Drive, Northcote, Auckland, 0627 New Zealand

**Keywords:** Bibliometrics, Critical care nurses, Intensive care unit, Text analytics, Wellbeing

## Abstract

**Background:**

Intensive care nurse wellbeing is essential to a healthy healthcare workforce. Enhanced wellbeing has widespread benefits for workers. Bibliometrics enables quantitative analysis of bourgeoning online data. Here, a new model is developed and applied to explore empirical knowledge underpinning wellbeing and intensive care nurse wellbeing in terms of size and impact, disciplinary reach, and semantics.

**Methods:**

Mixed methods bibliometric study. Firstly, a new model coined ‘iAnalysis’ was developed for the analysis of published data. Secondly, iAnalysis was applied in two studies to examine wellbeing and ICU nurse wellbeing. Study one explored data from a title search with search terms [wellbeing OR well-being], identifying 17,543 records with bibliographic data. This dataset included 20,526 keywords. Of the identified records, 10,715 full-text manuscripts were retrieved. Study two explored data from a topic search with search terms [(intensive OR critical) AND (nurs*) AND (wellbeing OR well-being)], identifying 383 records with bibliographic data. This dataset included 1223 author keywords. Of the identified records, 328 full-text manuscripts were retrieved.

**Results:**

Once data were collected, for *size and impact,* WoS Clarivate Analytics™ and RStudio™ were used to explore publication dates, frequencies, and citation performance. For *disciplinary reach,* RStudio™ (with the Bibliometrics™ package & Vosviewer™ plugin) was used to explore the records in terms of country of publication, journal presence, and mapping of authors. For *semantics*, once the bibliographic data was imported to RStudio™ (with the Bibliometrics™ package & Vosviewer™ plugin) keyword co-occurrences were identified and visualised. Full-text manuscripts were imported to NVivo™ to explore word frequencies of both the keywords and full-text manuscripts using the word frequency search. For both studies, records were predominantly published in the past 5 years, in English language, and from USA. The highest keyword co-occurrence for study one was “health and well-being”, and for study two, “family and model”.

**Conclusions:**

Terms commonly associated with ‘illbeing’, as opposed to ‘wellbeing’, were highly prevalent in both study datasets, but more so in intensive care nurse wellbeing data. Intensive care nurse wellbeing was virtually absent in this literature. The iAnalysis model provided a practice-friendly tool to explore a large source of online published literature.

**Electronic supplementary material:**

The online version of this article (10.1186/s12912-019-0343-1) contains supplementary material, which is available to authorized users.

## Background

### Wellbeing

The wellbeing of intensive care (ICU) nurses is a fundamental component of healthcare workforce health. Opportunities to enhance the working lives of nurses are increasingly evident in the literature [1–5]. These opportunities are coupled with ongoing calls for more rigour in the research process to provide evidence of the effectiveness and sustainability of interventions to improve wellbeing [4, 5].

Wellbeing has a broad range of definitions, such as, “the balance point between an individual’s resource pool and the challenges faced” ([6], p., 230), and “the combination of feeling good and functioning effectively” ([7], p., 139). Increasingly the literature is pointing to wellbeing being a rich and multi-faceted construct [8]. Work wellbeing has equally varied and predominantly Western theoretical models, views, and definitions [9–13]. Whilst no one specific feature is evident across these models, variations of the element ‘relationships’ or ‘social connections’ are apparent in most theoretical models. No models specific to nurse wellbeing nor nurse work wellbeing were identified in the literature despite considerable research in the areas of work engagement, job demands, and personal resources (e.g., [14]). A systematic review of longitudinal studies that investigated employee wellbeing found the majority of the 40 identified studies focussed on illbeing, or the “negative side” of employee wellbeing [15]. For example, over half of the studies focused on burnout. This focus on illbeing is consistent with the literature on the assessment of nurses [16–26].

Wellbeing is increasingly prevalent in policy and legislation both nationally [27] and internationally [28]. Measuring how this policy and legislation translates into the working lives of ICU nurses is an ongoing challenge for key stakeholders including nurses, employers, professional organisations, and government bodies. Enhanced workplace wellbeing in healthcare has widespread benefits including improved performance, engagement, patient satisfaction, and lower turnover costs [29–35]. However, little is known about the structure of the empirical foundations of work wellbeing for ICU nurses. Mapping the structure of ICU nurse wellbeing evidence base will create unique opportunities to identify the patterns and trends in relation to the semantics, literary reach, and impact of the literature. The synthesis of information systems knowledge has led to a range of literature review methods, such as narrative, descriptive, critical, scoping, and systematic [36]. What these methods do not provide is a model for the exploration of extant literature in terms of bibliometrics and text analysis.

### Information metrics (iMetrics)

Online publication processes have given rise to opportunities for quantitative analysis of academic publishing. This quantitative analysis has various forms under the umbrella of *information metrics* (iMetrics) [37]. One of the most common forms of iMetrics is bibliometrics. Bibliometrics is purported to provide a “dynamic view of concepts and semantics” ([38], p. 1315), and enables quantitative analysis of large amounts of data. Bibliometrics most common use to date has been characterising scientific output and citations of researchers (for example, the h-index), journals (for example, the Journal Impact Factor), and article impact [39]. Debate and controversy exist within these key areas [40], with common definitions and measurement indicators still evolving [41].

Bibliometric techniques are also being used for opinion mining and sentiment analysis [42] and to explore concepts such as nursing identity and patient-centeredness [43]. Using computation text analysis, Bell, Campbell and Goldberg [43] identified a disconnect between the two concepts, suggesting the text analysis provided a “bird’s eye view” and a valuable “scoping method” (p. 14), as opposed to the depth of analysis in, for example, a systematic review. In another example, Goodwin, VanDyne, Lin and Talbert [44] examined the use of data-mining techniques with patients’ clinical records. Whilst authors noted the potential for clinical data entry errors to bias findings (“garbage-in … garbage-out”, p. 387), Goodwin, VanDyne, Lin and Talbert [44] acknowledge the value of data-mining methods in building knowledge. Discovering knowledge from these textual databases requires the extraction of meaningful data [45]. Novel tools are needed for investigating concepts and developing conceptual clarity before individuals, teams, and organisations (such as researchers, healthcare professionals, legislative bodies, and policy analysts) make recommendations for change. To achieve this meaningful data extraction, many different methods have been proposed [46–48]. However, no model has yet been identified that draws together the proposed methods and translates these into a practice-friendly model that can be applied to a range of clinical questions. The current research draws on the early approach to Knowledge Discovery in Databases (KDD) of Fayyad [49] to develop a new model for exploring online data in terms of bibliometrics and text analysis. With the exponential growth in online publishing, practice-friendly tools are needed to identify and extract relevant data to inform nursing research and practice advancements.

### Research objectives

The aim of this research was to explore the size and impact, disciplinary reach, and semantics of wellbeing (Study 1), and then, more specifically, ICU nurse wellbeing (Study 2). To achieve this, firstly, the iAnalysis model is developed. Secondly, the iAnalysis model is applied to two bodies of online data. Such a process will create new knowledge in relation to the concepts of both wellbeing, and ICU nurse wellbeing.

## Methods

### iAnalysis model development

**Fig. 1:**
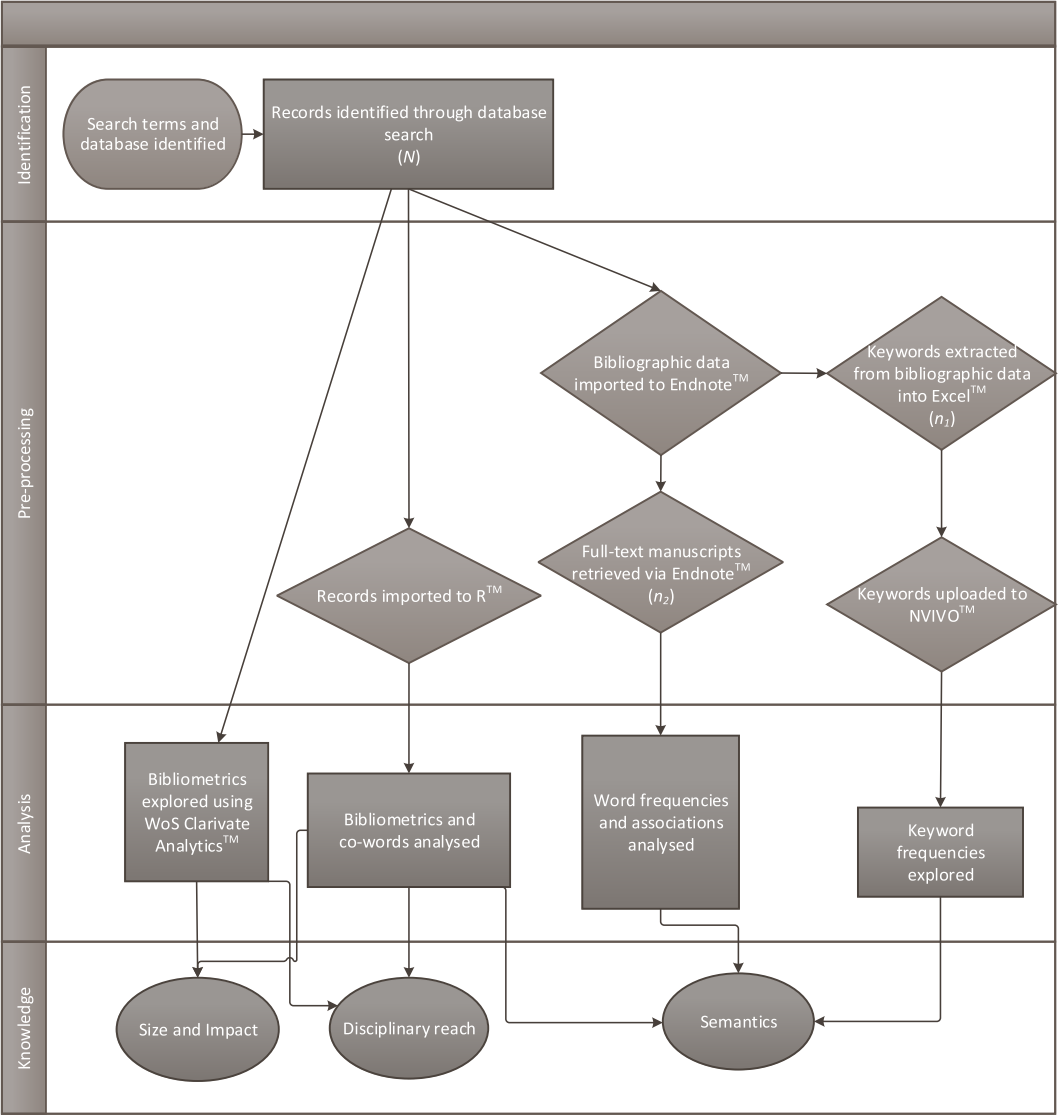
iAnalysis model. *Note*: *N* = all records; *n*_1_ = sample of keywords; n_2_ = sample of full-text manuscripts; WoS = Web of Science

The KDD process and co-word analysis approaches (e.g., [49]) were used to examine the nature of relationships and structure of knowledge of the wellbeing and ICU nurse wellbeing literature. This study employed research tools generally available in academic settings, rather than specialised tools and program plug-ins, to develop a flexible and adaptable mixed methods approach to explore online published literature. Thus, the model is intended to be generalisable to other researchers across a variety of practice-based fields. The adapted text analysis method, that we have called iAnalysis, was then applied to two datasets, 1) wellbeing and 2) ICU nurse wellbeing. Application of the model explored the two datasets in terms of 1) size and impact, 2) disciplinary reach, and 3) semantics. The iAnalysis model is depicted in Fig. 1.

### Procedure

#### Data collection

The data collection (identification), pre-processing, and analysis method are illustrated in Fig. 1. The Web of Science™ (WoS) advanced search engine was used to retrieve the dataset. Records retrieved in the WoS Core Collection™ title search were explored using seven tools described in the following analysis section.

#### Identification

Given the vast number and variable quality of publications available through online publishers and databases, clarity of the inclusion criteria for this study was essential. A wide range of web-based databases are available. For this study, the WoS was selected primarily because the journals in the database are in the Science Citation Index (SCI) and the Social Science Citation Index (SCCI). Further, the WoS makes detailed bibliometric analysis possible. For wellbeing, the key search terms in the WoS search engine included: *wellbeing* OR *well-being*. The article title search was conducted on the 16 February 2018 for all years up until 2017. No language limiters were applied. For ICU nurse wellbeing, the key search terms in the WoS search engine included: critical OR intensive, AND nurs*, AND wellbeing OR well-being. The article search was conducted on 14 February 2018 with the date limiter from 1900 to 2017 applied. No language limiters were applied. The broadest search category in WoS, “Topic Search”, was selected.

#### Pre-processing

Both the wellbeing and ICU nurse wellbeing datasets (*N*) comprised all bibliographic data from this initial search (e.g., authors, titles, journals, publication dates, citations). A subset of this primary dataset were the full-text manuscripts retrieved (*n*_*2*_). The primary dataset was explored with WoS Clarivate Analytics™ (a WoS data analysis software), imported to Endnote™ (a bibliographic referencing software), and to Excel™ (Microsoft office program). Both author keywords (*n*_*1*_) and the full text manuscripts (*n*_*2*_) were imported to NVivo™ (qualitative data software; QRS International, Victoria AU). Records were also imported to the open source statistical program RStudio™ with the Bibliometrics™ package [50] and the Vosviewer™ plugin (open source data analysis software).

#### Analysis

Data analysis focused on 1) size and impact, 2) disciplinary reach, and 3) semantics. Once the data was collected, for *size and impact,* WoS Clarivate Analytics™ and RStudio™ were used to explore publication dates, frequencies, and citation performance (i.e., h-index or Hirsh factor; [51]). For *disciplinary reach,* RStudio™ (with the Bibliometrics™ package & Vosviewer™ plugin) was used to explore the records in terms of country of publication, journal presence, and mapping of authors. For *semantics*, once the bibliographic data was imported to RStudio™ (with the Bibliometrics™ package & Vosviewer™ plugin) keyword co-occurrences were identified and visualised. Full-text manuscripts were imported to NVivo™, and common stopwords were excluded from the analysis (e.g., method, results). NVivo™ was used to explore word frequencies of both the keywords (*n*_*1*_) and full-text manuscripts (*n*_*2*_) using the word frequency search for the 1000 most frequent words with a minimum length of 5 letters.

## Results

### Study one: wellbeing

The WoS Core Collection™ topic search identified 17,543 records with bibliographic data (*N*). This dataset included 20,526 keywords (*n*_*1*_). Of the identified records (*N*), 10,715 full-text manuscripts were retrieved (*n*_*2*_).

#### Size and impact

The retrieved records (*N* = 17,543) had an average number of citations per record of 21.9 (highest individual = 7880). Greater than 50% of all records were published in the past 5 years and the earliest record was from the year 1917. The records covered a range of health-related areas, with the highest number of citations for an individual manuscript [52] being 7880 citations (437 per annum [pa]).

#### Disciplinary reach

The most frequent journals represented were *Social Indicators Research* (*n* = 605)*, Journal of Happiness Studies* (*n* = 246)*,* and *Personality and Individual Differences* (*n* = 201)*, Social Science and Medicine* (*n* = 159)*,* and *Aging and Mental Health* (*n* = 108). Eight authors had published on more than 30 occasions (Deiner, E; Casas, F; Oishi, S; Cummins, R; Ryff, C; Kaplan, R; Ryan, R; Shek, D), the most frequently observed author was Ed Diener with 79 publications. The most frequently appearing country of research was USA (*n* = 5307), followed by England (*n* = 1783), Australia (*n* = 1380), Canada (*n* = 1005), and Germany (*n* = 630) (source: *R*™). More than 95% of publications were in the English language (96%), followed by Spanish (1%) and German (1%) (source: WoS Clarivate Analytics™ & RStudio™).

#### Semantics

**Fig. 2:**
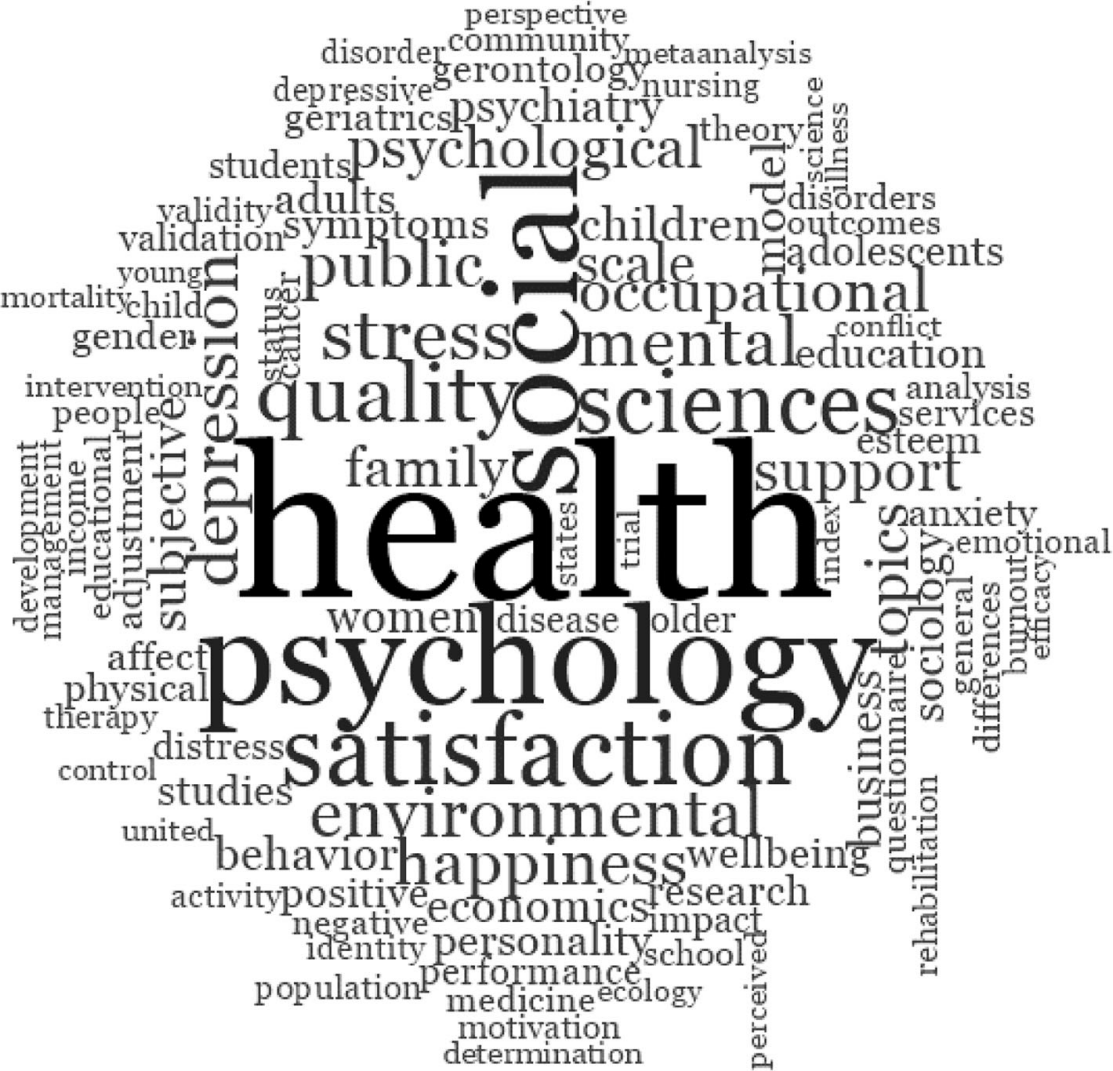
Study one exploratory keyword cloud for wellbeing. *Note*: Figure from NVivo’s™ word frequency query illustrating the 1000 most frequent exact word matches with minimum length of five characters. Word size represents frequency

Firstly, keywords of the bibliographic dataset (*n* = 20,526) were explored. The 10 most frequently appearing terms (excluding common stopwords) included “health” (10,387), “psychology” (6181), “social” (5926), “satisfaction” (4199), “sciences” (3585), “quality” (3404), “stress” (2870), “mental” (2616), “environmental” (2519), and “depression” (2513). The most frequent keywords are illustrated in a word cloud (see Fig. 2).

**Fig. 3:**
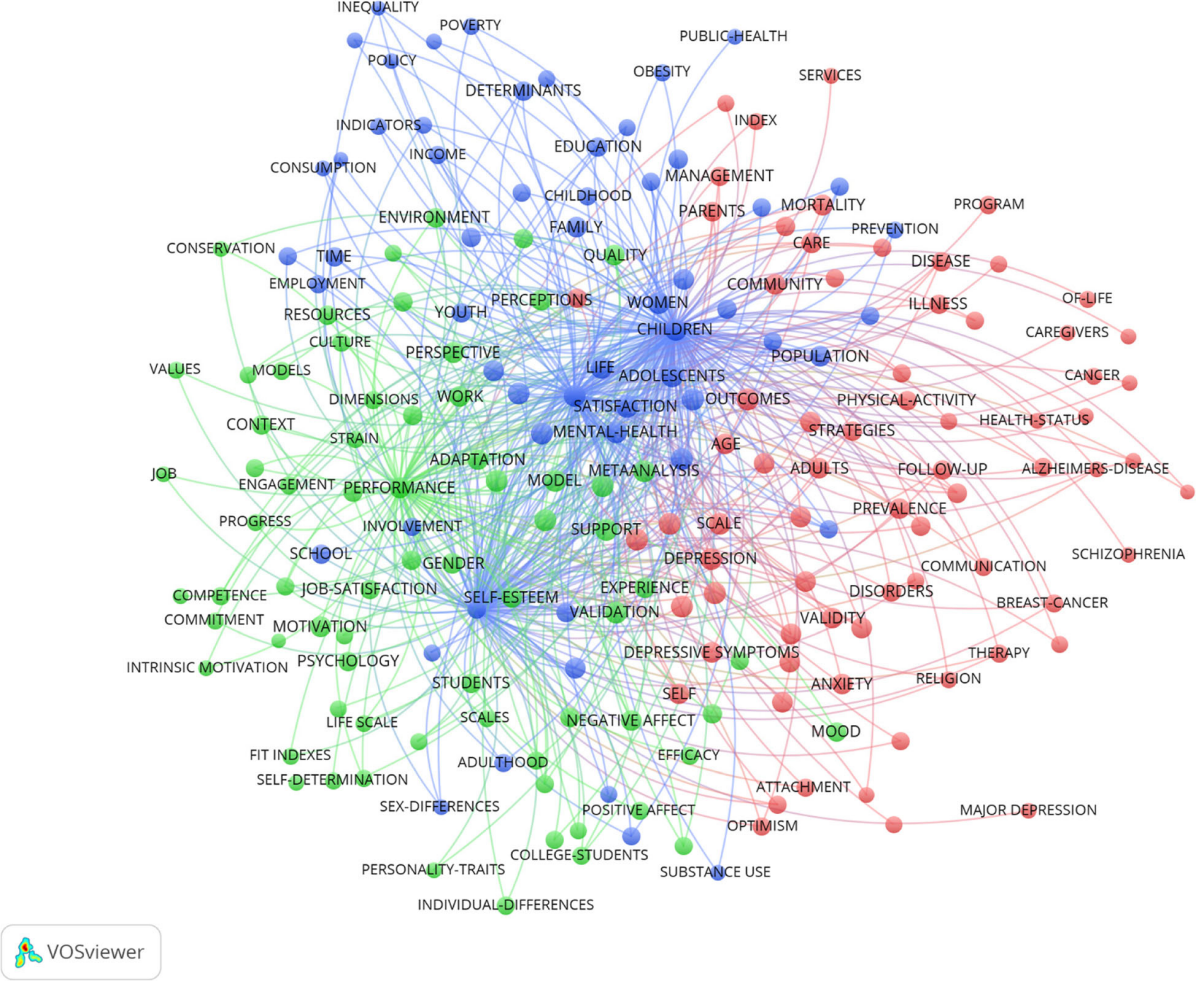
Study one keyword co-occurrences for wellbeing. *Note*: Visualisation from *VOSviewer*™ (*n* = 200). The size of the label and the circle of an item indicates the frequency of the keyword. The higher the frequency the larger the size. The colour of an item indicates the cluster the keywords belong to. Distances between keywords indicates relatedness of keywords in terms of co-occurrence links. Closer terms indicate closer relatedness. The strongest co-occurrences are represented by lines

The highest keyword co-occurrence was “health and well-being” (*n* = 562) followed by “happiness and subjective well-being” (*n* = 412), “happiness and well-being” (*n* = 390), “satisfaction and wellbeing” (*n* = 352), “quality of life and wellbeing” (*n* = 323), “quality-of-life and quality of life” (*n* = 299), “mental health and well-being” (*n* = 298), “life and well-being” (*n* = 290), “stress and well-being” (*n* = 272), and “depression and well-being” (*n* = 256) (see Fig. 3).

Secondly, the full-text manuscripts (*n* = 10, 715) were explored. The most frequently presenting words were identified, and are presented in terms of weighted percentage (see Additional file 1). Examples included “health” (*n* = 335,637), “social” (*n* = 280,696), “children” (*n* = 144,235), “psychological” (*n* = 137,772), “satisfaction” (*n* = 133,768), “positive” (*n* = 129,053), “support” (*n* = 112,173), “family” (*n* = 109,738), “women” (*n* = 92,704), and “mental” (*n* = 91,586). “Family” and “support” were explored in further detail using a text search query and word tree. Firstly, “family” presented in relation to “support”, “conflict”, and “responsibilities”. “Support” presented as “professional” and “organisational” support within the workplace, and “family” and “social” support. The manuscripts were also searched for additional terms commonly associated with wellbeing in the literature. The following terms were identified: “relationships” (*n* = 57,158), “happiness” (*n* = 53,849), “meaning” (*n* = 23,692), “engagement” (*n* = 20,359), “motivation” (*n* = 17,182), “purpose” (*n* = 16,660), and “achievement” (*n* = 11,480). Of note, terms commonly associated with illbeing were also highly frequent, such as “stress” (*n* = 74,014), “depression” (*n* = 65,383), and “anxiety” (*n* = 41,501).

### Study two: ICU nurse wellbeing

This subset of wellbeing was identified in a new systematic search. The WoS Core Collection™ topic search identified 383 records with bibliographic data (*N*). This dataset included 1223 author keywords (*n*_*1*_). Of the identified records (*N*), 328 full-text manuscripts were retrieved (*n*_*2*_).

#### Size and impact

The retrieved records (*N* = 383) had an h-index of 37, with an average number of citations per item of 14.9 (highest individual = 342), sum of times cited 5731, and citing articles 5496. Almost 50% of all records were published in the past 5 years and the earliest record was from the year 1992. The records covered a range of health-related areas such as psychological wellbeing, emotion regulation, moods and judgements, and models of wellbeing. The highest number of citations for an individual manuscript [52] was 342 citations (24 per annum [pa]).

#### Disciplinary reach

The most frequent journals represented were *Journal of Advanced Nursing* (*n* = 29)*, Journal of Clinical Nursing* (*n* = 25)*, Nursing in Critical Care* (*n* = 9)*, International Journal of Nursing Studies* (*n* = 8), *Critical Care Medicine* (*n* = 7)*,* and *Intensive and Critical Care Nursing* (*n* = 7). Ten authors had published on more than two occasions (Kleinpell, R.; Good, V.; Gozal, D.; Moss, M.; Sessler, C.; Jackson, D.; Le Blanc, P.; Lee, S.; Mixer, S.; Schaufeli, W.), the most frequently observed author was Ruth Kleinpell with 6 publications. The most frequently appearing country of research was USA (*n* = 114), followed by Australia (*n* = 32), England (*n* = 32), Canada (*n* = 25), and Sweden (*n* = 19) (source: *R*™). More than 90% of publications were in the English language (96%), followed by German (2%) (source: WoS Clarivate Analytics™).

#### Semantics

**Fig. 4:**
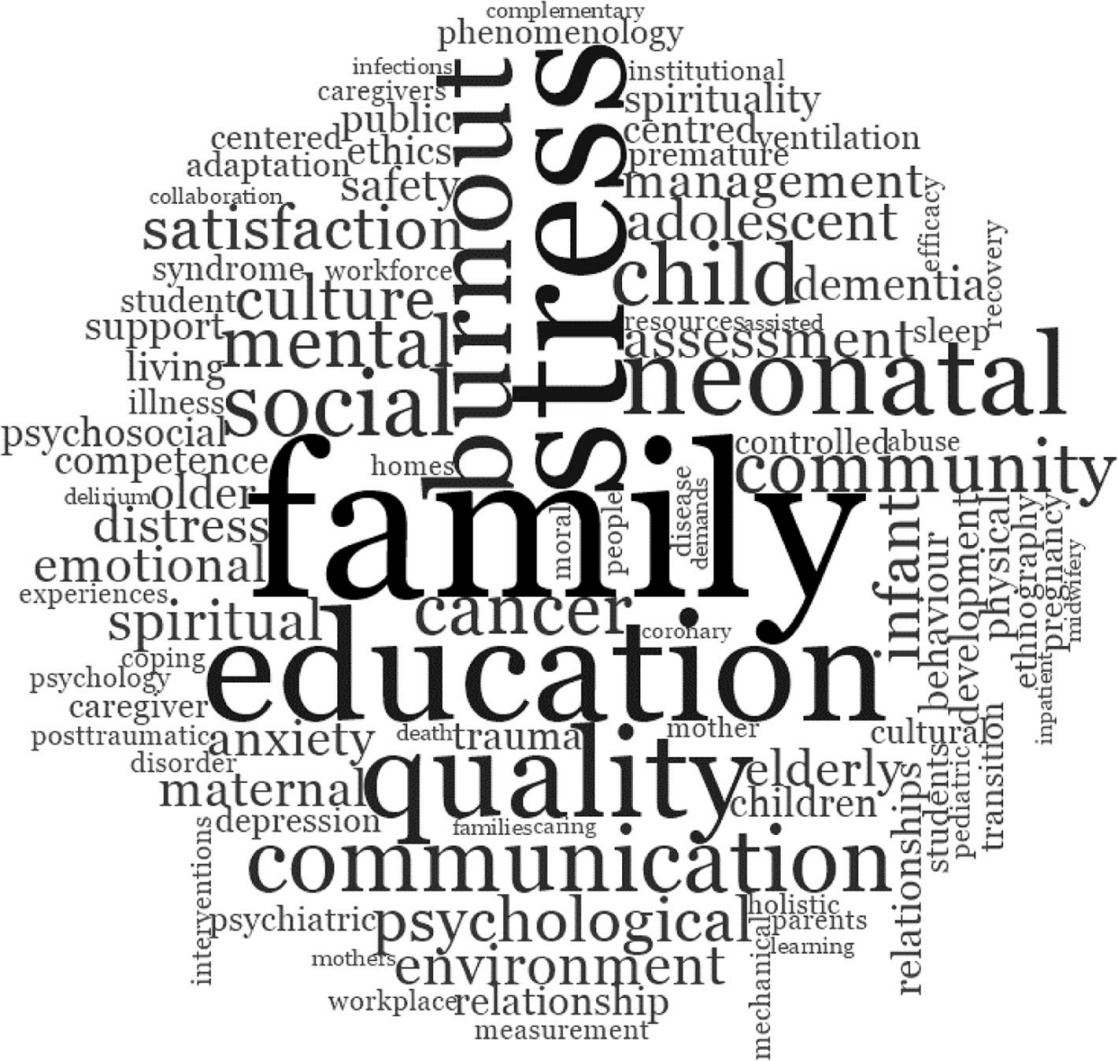
Study two exploratory keyword cloud for ICU nurse wellbeing. *Note*: Figure from NVivo’s™ word frequency query illustrating the 1000 most frequent exact word matches with minimum length of five characters less common stop words. Word size represents frequency

Firstly, author keywords of the bibliographic dataset (*n* = 1223) were explored. The 10 most frequently appearing terms (excluding stopwords such as “unit”, “intensive”, “critical”, “nurse”, “units”) included “family” (*n* = 38), “stress” (*n* = 31), “education” (*n* = 27), “quality” (*n* = 22), “burnout” (*n* = 21), “neonatal” (*n* = 20), “communication” (*n* = 16), “social” (*n* = 16), “child” (*n* = 15), and “cancer” (*n* = 13). The term “wellbeing” (“well being” OR “well-being” OR “wellbeing”) appeared on 24 occasions in the keywords in association with the words “psychological” (i.e., “psychological well-being”), “spiritual”, “subjective”, “caregiver”, and “emotional”. The most frequent keywords are illustrated in a word cloud (see Fig. 4).

The ICU nurse wellbeing keywords were also searched for additional terms commonly associated with wellbeing in the literature. The text search identified the following terms and frequencies, “satisfaction” (*n* = 11), “teamwork” (*n* = 1), “relationships” (*n* = 7), and “engagement” (*n* = 2).

**Fig. 5:**
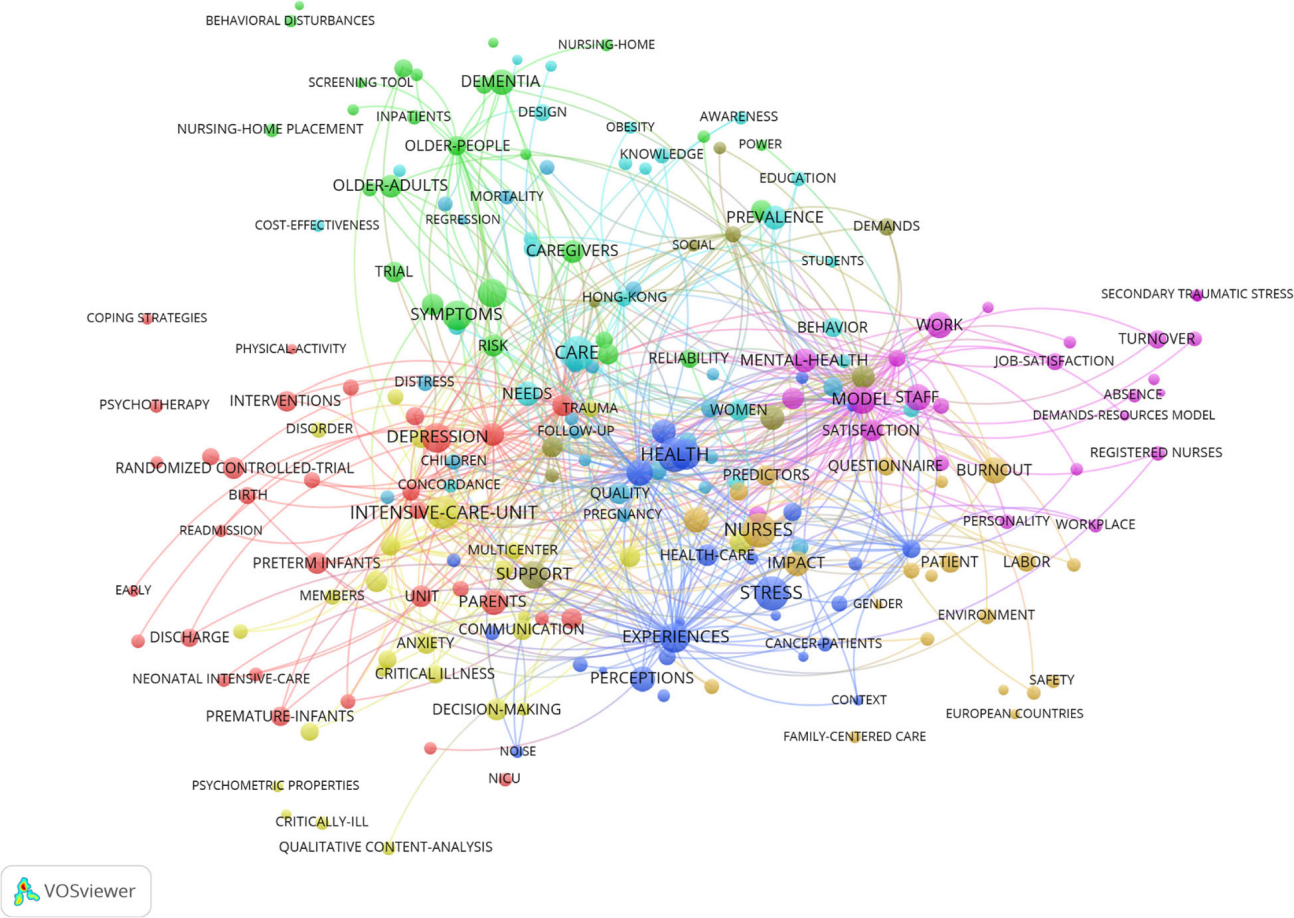
Study two keyword co-occurrences for ICU nurse wellbeing. *Note*: Visualisation from *VOSviewer*™ (*n* = 200). The size of the label and the circle of an item indicates the frequency of the keyword. The higher the frequency the larger the size. The colour of an item indicates the cluster the keywords belong to. Distances between keywords indicates relatedness of keywords in terms of co-occurrence links. Closer terms indicate closer relatedness. The strongest co-occurrences are represented by lines

The top 20 keyword co-occurrences all included the term “model”. Co-term examples included: “family” (i.e., “model & family”), “stress”, and “intervention” (see Fig. 5).

Keywords potentially related to wellbeing (as opposed to illbeing) occurring in the top 200 co-occurrences, included “satisfaction”, “mental health”, and “quality of life”.

Secondly, the full-text manuscripts (*n* = 328) were explored. The most frequently presenting words were identified, and are presented in terms of weighted percentage (see Additional file 1). Examples included “health” (*n* = 11,298), “family” (*n* = 4,561), “support” (*n* = 3,190), “social” (*n* = 3,185), “stress” (*n* = 2,986), “people” (*n* = 2,492), “information” (*n* = 2,488), “quality” (*n* = 2,459), “experience” (*n* = 2,422), “children” (*n* = 2,332), “education” (*n* = 2,109), and “management” (*n* = 1,998). Family and support were explored in further detail using a text search query and word tree. Firstly, ‘family’ presented as the nurses’ relationships with patients’ families in ICU, (e.g., connections, presence, satisfaction, & communication). Secondly, ‘support’ presented as logistics, organisational, management, senior staff, communication, mentorship, and for patient and family. Searching for additional terms commonly associated with wellbeing identified: “relationships” (*n* = 1,079), “happiness” (*n* = 0), “meaning” (*n* = 768), “engagement” (*n* = 415), “motivation” (*n* = 219), “purpose” (*n* = 492), and “achievement” (*n* = 0). Of note, terms commonly associated with illbeing were also highly frequent, such as “stress” (*n* = 2,986), “anxiety” (*n* = 1,240), “depression” (*n* = 1,069), and “burnout” (*n* = 1,473).

The word frequencies in the full-text manuscripts of studies 1 and 2 were then compared to identify which of the most commonly presenting words occurred in both studies (see Additional file 1). Examples of commonly co-occurring words included “health”, “social”, “children”, “psychological”, “satisfaction”, “positive”, “support”, “family”, “wellbeing”, “women”, and “mental”. Of note, “stress”, “depression”, and “anxiety” were terms present in the top 100 most frequently co-occurring terms in these full-text manuscripts.

## Discussion

### Mapping wellbeing

The salience of illbeing, specifically stress, anxiety, depression and burnout, in wellbeing research was evident in this iAnalysis. This salience was particularly prominent in the ICU nurse wellbeing literature. The iAnalysis sought to explore the empirical knowledge underpinning wellbeing and intensive care nurse wellbeing, yet the presence of illbeing occurred in semantic analyses of key words and co-terms. Such a result is likely due to a relatively recent paradigm shift to health promotion approaches, which is yet to translate into, and be observed in, the published literature. The semantics analysis identified several key elements thought to be indicative of wellbeing, such as meaning, engagement, motivation, purpose, positive emotions (e.g., happiness) and achievement. Whilst these elements were evident in the iAnalysis, they were under-represented in the results in comparison to terms such as stress, depression, anxiety and burnout.

Research focusing on illbeing has an important place in terms of healthcare. However, there is an increasing movement to balance this illbeing research with research related to wellbeing, particularly in relation to health promotion and prevention. Peterson [53] suggested “what is good about life is as genuine as what is bad and therefore deserves equal attention” (p. 4). Whilst this iAnalysis maps an image of illbeing, there is considerable work underway to create a balance [54–57]. The growth in the field of positive psychology is just one area where this is evident, promoting the scientific exploration of what makes life worth living [58]. With the recent adoption of this balanced approach, future application of iAnalysis will likely identify new and emerging trends in the literature with an increased focus on wellbeing.

### iAnalysis model

The iAnalysis model provided a practice-friendly tool to explore a large source of online, published literature. We were able to obtain a strong understanding of the existing research in relation to ICU nurse wellbeing. The iAnalysis model will have widespread benefits in future applications by researchers. Three examples of model benefits for researchers include 1) mapping of the structure of concepts prior to conducting a systematic or scoping review, 2) reviewing trends in research over time, and 3) identifying opportunities for future research.

### Limitations

This study has identified a distinct focus on illbeing in this collection of wellbeing literature. We have also described this literature base by reporting the *h*-index, average citations, and most frequently occurring journals publishing in this area. However, although widely used, the *h-*index has been termed “counter-intuitive”, providing inconsistent results when aggregating publication and citation statistics into a single number [59]. Similarly, counting citations is also problematic and error-prone due to the complete-normalised counts (see [60]). Thus, care needs to be taken in this interpretation of the findings. The iAnalysis method lacked elements of both specificity and sensitivity. In terms of specificity and sensitivity, future researchers will need to evaluate the cost versus benefit of either a more specific search potentially omitting relevant publications or a broader search increasing the potential for more irrelevant publications. There is likely bias from several sources, for example, publication bias, bias in source selection, and bias in publication language. These biases could be reduced by broadening the search to include of grey literature, drawing from wider sources, and adding other language interpretations for key search terms. Also, the technical problems related to counting publications, such as normal probability curves of publication growth rates and partial capturing of research publications by databases [61] are a further limitation. For example, the use of the WoS database is a limitation in itself, given the low number of nursing publications included [62]. Thus, the use of this method in construct and content validity in the future development of measures and/or interventions associated with ICU nurse wellbeing needs to be both considered and guarded. Future research using the iAnalysis would benefit from expanding on the databases searched. Broadening the search and further testing of the iAnalysis across databases would provide opportunities for model development. Nevertheless, this study provides insight into the structure of both the wellbeing, and ICU nurse wellbeing literature in relation to size and disciplinary reach, impact, and semantics. Investigating ICU nurses’ conceptions of wellbeing is an important next step towards enhancing the work life of ICU nurses.

## Conclusions

This research has developed and applied a new ‘iAnalysis’ model in two studies that integrated several readily available online research resources. Using the model, we analysed two bodies of online data from WoS searches and mapped the size and disciplinary reach, impact, and semantics of the wellbeing, and ICU nurse wellbeing datasets obtained. The iAnalysis provided four key contributions to the literature. Firstly, it demonstrated conceptions of illbeing were strongly represented in wellbeing and intensive care nurse wellbeing literature highlighting the opportunity for further research to balance these conceptions. Secondly, the map of ICU nurse wellbeing enhanced our understanding of the construct and supports the development of programmes to improve the work wellbeing of ICU nurses. Thirdly, the iAnalysis provides a practice-friendly tool to explore a large source of online published literature. Given the prolific and exponential growth of publications in peer-reviewed journals, this tool provides new ways to investigate these publications on a broader scale. Finally, the iAnalysis is a valuable model for text mapping that could be applied to a range of clinical questions, exploring these questions in new and unique ways.

## Additional file

Additional file 1: Table of full-text 100 most frequent words and co-occurring terms across studies 1 and 2. Additional File 1 presents a table of the 100 most frequent words and co-occurring terms for wellbeing and intensive care nurse wellbeing from the full-text analysis. (DOCX 22 kb)

## Abbreviations

ICU: Intensive care unit
WoS: Web of Science
