# Supplementary material for: Bibliometric mapping of intensive care nurses’ wellbeing: development and application of the new iAnalysis model
Source: BMC Nurs. 2019 Jun 3;18:21. doi: 10.1186/s12912-019-0343-1 (PMC6547532; doi:10.1186/s12912-019-0343-1)
Supplement: Supplementary file 1 — Table of full-text 100 most frequent words and co-occurring terms across studies 1 and 2. Additional File 1 presents a table of the 100 most frequent words and co-occurring terms for wellbeing and intensive care nurse wellbeing from the full-text analysis. (DOCX 22 kb) [file 12912_2019_343_MOESM1_ESM.docx]

**Additional file 1. Table of full-text 100 most frequent words and co-occurring terms across studies 1 and 2.**

| **Wellbeing** | **Wellbeing Count** | **Wellbeing Weighted Percentage (%)** | **Intensive care nurse wellbeing** | **Intensive care nurse wellbeing Count** | **Intensive care nurse wellbeing Weighted Percentage (%)** | **Co-occurring terms from study 1 & study 2** |
| --- | --- | --- | --- | --- | --- | --- |
| health | 335637 | 0.56 | health | 11298 | 0.71 | health |
| social | 280696 | 0.47 | nursing | 10085 | 0.63 | social |
| study | 221752 | 0.37 | nurses | 7971 | 0.50 | study |
| research | 179063 | 0.30 | study | 7592 | 0.48 | research |
| journal | 148864 | 0.25 | patients | 6361 | 0.40 | journal |
| children | 144235 | 0.24 | patient | 5691 | 0.36 | children |
| psychological | 137772 | 0.23 | research | 5345 | 0.34 | psychological |
| satisfaction | 133768 | 0.22 | journal | 4696 | 0.30 | satisfaction |
| positive | 129053 | 0.22 | family | 4561 | 0.29 | positive |
| support | 112173 | 0.19 | nurse | 4211 | 0.26 | support |
| family | 109738 | 0.18 | practice | 3401 | 0.21 | family |
| model | 109343 | 0.18 | critical | 3302 | 0.21 | model |
| people | 106899 | 0.18 | support | 3190 | 0.20 | people |
| group | 100033 | 0.17 | social | 3185 | 0.20 | group |
| scale | 96906 | 0.16 | stress | 2986 | 0.19 | scale |
| table | 94163 | 0.16 | clinical | 2981 | 0.19 | table |
| wellbeing | 93988 | 0.16 | group | 2832 | 0.18 | wellbeing |
| studies | 93948 | 0.16 | participants | 2716 | 0.17 | studies |
| results | 93751 | 0.16 | staff | 2579 | 0.16 | results |
| women | 92704 | 0.16 | intensive | 2506 | 0.16 | women |
| effects | 92305 | 0.15 | people | 2492 | 0.16 | effects |
| mental | 91586 | 0.15 | information | 2488 | 0.16 | mental |
| among | 90059 | 0.15 | quality | 2459 | 0.15 | among |
| variables | 88360 | 0.15 | studies | 2426 | 0.15 | variables |
| related | 87815 | 0.15 | experience | 2422 | 0.15 | related |
| participants | 87352 | 0.15 | hospital | 2365 | 0.15 | participants |
| negative | 86954 | 0.15 | analysis | 2360 | 0.15 | negative |
| analysis | 86818 | 0.15 | children | 2332 | 0.15 | analysis |
| relationship | 85498 | 0.14 | intervention | 2330 | 0.15 | relationship |
| child | 85194 | 0.14 | based | 2324 | 0.15 | child |
| psychology | 84637 | 0.14 | emotional | 2203 | 0.14 | psychology |
| subjective | 82744 | 0.14 | related | 2153 | 0.14 | subjective |
| level | 82736 | 0.14 | education | 2109 | 0.13 | level |
| sample | 79757 | 0.13 | medical | 2093 | 0.13 | sample |
| university | 79199 | 0.13 | university | 2015 | 0.13 | university |
| higher | 78978 | 0.13 | management | 1998 | 0.13 | higher |
| physical | 77119 | 0.13 | child | 1970 | 0.12 | physical |
| quality | 76002 | 0.13 | development | 1966 | 0.12 | quality |
| measures | 74022 | 0.12 | needs | 1893 | 0.12 | measures |
| stress | 74014 | 0.12 | women | 1889 | 0.12 | stress |
| school | 73931 | 0.12 | mental | 1887 | 0.12 | school |
| reported | 72042 | 0.12 | physical | 1861 | 0.12 | reported |
| income | 71940 | 0.12 | among | 1845 | 0.12 | associated |
| associated | 71876 | 0.12 | table | 1842 | 0.12 | found |
| found | 71459 | 0.12 | important | 1796 | 0.11 | development |
| development | 70578 | 0.12 | knowledge | 1793 | 0.11 | however |
| however | 70030 | 0.12 | using | 1773 | 0.11 | using |
| using | 69403 | 0.12 | control | 1768 | 0.11 | effect |
| effect | 69367 | 0.12 | results | 1766 | 0.11 | years |
| years | 68996 | 0.12 | factors | 1752 | 0.11 | levels |
| levels | 68597 | 0.12 | years | 1748 | 0.11 | based |
| based | 68414 | 0.11 | members | 1747 | 0.11 | individual |
| individual | 67991 | 0.11 | model | 1741 | 0.11 | emotional |
| emotional | 65585 | 0.11 | psychological | 1728 | 0.11 | depression |
| depression | 65383 | 0.11 | reported | 1721 | 0.11 | control |
| control | 64726 | 0.11 | community | 1684 | 0.11 | education |
| education | 64565 | 0.11 | level | 1667 | 0.10 | different |
| different | 63169 | 0.11 | however | 1651 | 0.10 | status |
| status | 63094 | 0.11 | experiences | 1642 | 0.10 | three |
| three | 61884 | 0.10 | outcomes | 1618 | 0.10 | factors |
| factors | 61541 | 0.10 | treatment | 1618 | 0.10 | items |
| items | 61368 | 0.10 | process | 1588 | 0.10 | differences |
| differences | 61139 | 0.10 | found | 1572 | 0.10 | scores |
| scores | 60282 | 0.10 | review | 1530 | 0.10 | important |
| important | 60265 | 0.10 | relationship | 1524 | 0.10 | affect |
| affect | 59721 | 0.10 | scale | 1505 | 0.09 | groups |
| groups | 58409 | 0.10 | parents | 1485 | 0.09 | community |
| community | 58165 | 0.10 | burnout | 1473 | 0.09 | economic |
| economic | 57622 | 0.10 | environment | 1472 | 0.09 | relationships |
| relationships | 57158 | 0.10 | families | 1460 | 0.09 | individuals |
| individuals | 54842 | 0.09 | communication | 1442 | 0.09 | number |
| number | 54190 | 0.09 | cancer | 1435 | 0.09 | general |
| general | 53902 | 0.09 | services | 1435 | 0.09 | outcomes |
| happiness | 53849 | 0.09 | professional | 1434 | 0.09 | patients |
| outcomes | 53485 | 0.09 | coping | 1431 | 0.09 | personal |
| patients | 53400 | 0.09 | positive | 1386 | 0.09 | gender |
| personal | 53246 | 0.09 | different | 1378 | 0.09 | experience |
| gender | 51055 | 0.09 | effects | 1376 | 0.09 | change |
| experience | 50960 | 0.09 | personal | 1374 | 0.09 | measure |
| change | 50366 | 0.08 | impact | 1370 | 0.09 | within |
| measure | 50039 | 0.08 | within | 1370 | 0.09 | impact |
| within | 49279 | 0.08 | older | 1361 | 0.09 | perceived |
| personality | 48868 | 0.08 | individual | 1357 | 0.09 | older |
| impact | 48489 | 0.08 | satisfaction | 1349 | 0.08 | author |
| perceived | 47720 | 0.08 | healthcare | 1343 | 0.08 | symptoms |
| older | 47642 | 0.08 | evidence | 1342 | 0.08 | population |
| author | 47011 | 0.08 | students | 1342 | 0.08 | survey |
| symptoms | 46997 | 0.08 | symptoms | 1342 | 0.08 | human |
| population | 46875 | 0.08 | training | 1335 | 0.08 | signiﬁcant |
| survey | 46801 | 0.08 | levels | 1317 | 0.08 | lower |
| human | 46470 | 0.08 | design | 1296 | 0.08 | review |
| signiﬁcant | 45842 | 0.08 | three | 1287 | 0.08 | factor |
| lower | 45715 | 0.08 | general | 1284 | 0.08 | behavior |
| review | 45372 | 0.08 | associated | 1273 | 0.08 | problems |
| factor | 44693 | 0.08 | number | 1268 | 0.08 | students |
| behavior | 43504 | 0.07 | interventions | 1260 | 0.08 | total |
| problems | 43463 | 0.07 | assessment | 1250 | 0.08 | values |
| students | 43346 | 0.07 | anxiety | 1240 | 0.08 | activities |
| total | 41774 | 0.07 | infant | 1240 | 0.08 | anxiety |
